# Serotonin-Mediated Avoidance and Immune Suppression in *Caenorhabditis elegans* Exposed to Sodium Arsenite

**DOI:** 10.1155/bri/8485137

**Published:** 2025-09-04

**Authors:** Xinyu Li, Jiaxin Zhao, Xiaoying Liu, Honglian Zhao, Xiaobing Zhang, Yaqi Deng, Qi Wang, Wei Zou

**Affiliations:** Yunnan Provincial Key Laboratory of Public Health and Biosafety & School of Public Health, Kunming Medical University, Kunming 650500, China

**Keywords:** *Caenorhabditis elegans*, escape, immune, sodium arsenite

## Abstract

Pathogen avoidance behavior is widespread across the animal kingdom and plays an important role in animal survival in natural environments. As a free-living nematode, *Caenorhabditis elegans* is exposed to various microorganisms and toxic chemicals in the soil, including pathogenic bacteria and chemical toxins. *C. elegans* can effectively avoid pathogenic bacteria, thereby minimizing the risk of infection. However, it remains unclear whether it also exhibits avoidance behavior in response to toxic substances such as sodium arsenite. In this report, we found that as the sodium arsenite concentration in the environment increased, the arsenic content in *C. elegans* also increased, leading to an avoidance response. Further investigation revealed that deletion of the serotonin synthesis gene *tph-1* significantly suppressed this avoidance behavior. Moreover, the expression level of TPH-1 protein in ADF neurons was significantly upregulated. Our data indicate that *C. elegans* exhibits a serotonin-mediated behavioral avoidance response to sodium arsenite. Additionally, we observed that the immune system of *C. elegans* was suppressed following sodium arsenite exposure, likely as an adaptation to the adverse environment. This study provides new insights into the strategies of *C. elegans* in response to toxic environmental conditions.

**Summary**

• This study reveals the behavioral avoidance mechanism of *Caenorhabditis elegans* in response to the toxic substance sodium arsenite and its underlying neural regulatory mechanisms.
• We found that as the concentration of sodium arsenite in the environment increased, *C. elegans* exhibited significant avoidance behavior, which was regulated by the serotonin synthesis gene *tph-1*.
• Additionally, exposure to sodium arsenite suppressed the immune system of *C. elegans*, likely as an adaptive strategy to adverse environmental conditions.
• These findings not only enhance our understanding of the survival strategies of invertebrates in toxic environments but also provide new insights into the study of neuro-immune interactions, highlighting the importance of exploring the impact of environmental toxicity on organismal behavior.

## 1. Introduction

Arsenic is a naturally occurring metalloid, widely distributed in the earth's crust, often associated with pyrite, and readily dissociates from minerals into soil and water [1]. The average arsenic concentration in European soils is approximately 7.0 mg/kg, whereas in the soils of Uttar Pradesh, India, it ranges from 16 to 417 mg/kg. In Mexico's Lagunera region, arsenic concentration in soils ranges from 2215 to 2675 mg/kg [2]. In China, approximately 2.0 × 10^7^ ha of farmland are contaminated with heavy metals such as cadmium, arsenic, and lead, with an average arsenic concentration of 11.2 mg/kg in Chinese soils—roughly twice the global average [3].

Arsenic exposure commonly disrupts plant metabolism and adversely affects soil-dwelling animals. Chronic arsenic exposure is associated with cancer, diabetes, cardiovascular diseases, and reproductive defects in humans [4]. Arsenic biotransformation is primarily mediated by environmental microorganisms and involves As (V) reduction, As (III) oxidation, arsenic methylation, demethylation, and transport [5]. *C. elegans*, a free-living nematode inhabiting soil rich in microorganisms and chemicals, perceives diverse environmental cues through its ciliated sensory neurons. These neurons play significant roles in chemotaxis, mechanosensation, osmosensation, and thermotaxis [6]. *C. elegans* can distinguish and avoid pathogenic bacteria, a behavior critical for its survival, as has been documented extensively [7]. Arsenic exposure induces redox imbalance in *C. elegans*, leading to cellular damage and death. The toxicity of arsenic in *C. elegans* differs between its arsenite (As (III)) and arsenate (As (V)) forms, with As (III) being more toxic. Arsenic exposure causes significant behavioral changes in *C. elegans*, including reduced mobility and impaired chemotaxis. These behavioral changes may be associated with neurotoxicity, affecting the nematode's normal behaviors. Physiologically, arsenic exposure inhibits growth and development in *C. elegans*. Chronic exposure to high concentrations of arsenic decreases reproductive capacity and lifespan, impacting offspring viability and development. Arsenic's effects on *C. elegans* are multifaceted, including behavioral, physiological, and genetic changes [8]. How does *C. elegans* cope with this toxicity? It has evolved multiple mechanisms to combat arsenic toxicity, including gene regulation and enhanced antioxidant defenses. Upon infection with *Pseudomonas aeruginosa*, *C. elegans* exhibits pathogen avoidance behavior [9]. Would *C. elegans* exhibit similar avoidance behavior to protect itself from arsenic in soil environments?

Certain signaling pathways have been reported to modulate avoidance behavior. For instance, activation of AWB chemosensory neurons, which detect serratamolide W2 produced by pathogenic *Serratia marcescens*, triggers avoidance behavior [10]. The TAX-4/TAX-2 cGMP-gated channel, insulin receptor DAF-2, neuropeptide receptor NPR-1, and E3 ubiquitin ligase HECW-1 are involved in innate avoidance responses, while serotonin (TPH-1), TGF-β signaling ligands (DBL-1 and DAF-7), and insulin-like peptides (INS-6 and INS-7) regulate learned pathogen avoidance [11]. Recent studies indicate that miRNA miR-67 participates in avoidance behavior, where *P. aeruginosa* PA14 induces miR-67 expression in *C. elegans*, and miR-67(n4899) mutants show reduced avoidance of *P. aeruginosa* PA14 [12]. NPR-1, a G protein-coupled receptor, modulates pathogen resistance by regulating oxygen-dependent avoidance behavior. HECW-1, an E3 ubiquitin ligase, suppresses *P. aeruginosa* avoidance behavior in OLL mechanosensory neurons, affecting NPR-1 regulation [13]. Additionally, *C. elegans* raised in the presence of pathogenic bacteria learns to avoid bacterial odors, a behavior that requires the ADF chemosensory neuron, tryptophan hydroxylase TPH-1, command interneurons AVA, and TGF-β family protein DAF-7 secreted from ASJ neurons in response to bacterial metabolites [14]. When *C. elegans* encounters pathogenic bacteria, behavioral avoidance strategies are enhanced via the HECW-1/NPR-1 module if innate immunity is compromised, suggesting that GPCRs in neural circuits may integrate signals from the immune system to adapt to adverse environments [13].

Interestingly, in this study, we found that sodium arsenite exposure induces avoidance behavior in *C. elegans* as a strategy to cope with adverse conditions. Further analysis revealed that deletion of the serotonin synthesis gene *tph-1* significantly suppressed this avoidance behavior. Additionally, TPH-1 protein expression in ADF neurons was significantly upregulated. Our data indicate that *C. elegans* exhibits a serotonin-mediated avoidance response to sodium arsenite, providing a new perspective on how nematodes respond to toxic environments. These findings not only contribute to the understanding of arsenic's biological effects but also provide a critical model for assessing the impact of environmental pollution on biological systems. Future research could further explore the effects of different arsenic compounds on *C. elegans*, as well as their potential ecological consequences and applications. This knowledge is of great significance in understanding environmental pollution and its biological impacts.

## 2. Materials and Methods

### 2.1. Strains

The *C. elegans* N2 strain, *tph-1*(mg280) mutant, and strains expressing *tph-1*::GFP (SyIs49), *daf-16*::GFP, and *skn-1*::GFP were obtained from the *C. elegans* Genetic Center (CGC). All strains were cultured on nematode growth medium (NGM) plates with *Escherichia coli OP50* (OP50) at 20°C.

### 2.2. Sodium Arsenite Small Lawn Avoidance Assays

OP50 was inoculated into LB medium and grown overnight at 20°C. A solution of sodium arsenite with concentrations of 0.05, 0.1, 0.2 and 0.5 mM is configured. Sodium arsenite is purchased from Chengdu West Asia Reagent Co., Ltd. This mixture was added to the center of 15 mm NGM plates and dried. Fifty to one hundred L4 stage worms were placed on the OP50 lawn, and avoidance was assessed at 8, 12, and 24 h at 20°C. Avoidance index = (number of worms outside the lawn)/(total number of worms), with at least three replicates per experiment.

### 2.3. Tuning Rate Method in *C. elegans*

To measure the tuning rate index, approximately 20 young adult *C. elegans* were placed on a microscope slide containing a drop of sodium arsenite solution at concentrations of 0, 0.2, and 0.5 mM. The microscope focus was adjusted to observe the nematodes, and the number of body bends within 30 s was counted for each condition.

### 2.4. Measurement of Arsenic Content in *C. elegans*

Synchronous L4-stage *C. elegans* were collected by centrifugation and transferred to NGM-OP50 plates containing different concentrations of sodium arsenite with 4 replicates per concentration. After 24 h of exposure, the nematodes were washed with M9 buffer, weighed, and digested in conical flasks. The digested samples were transferred to 25 mL volumetric tubes. Arsenic standard solutions were prepared by diluting a 1000-μg/mL single-element arsenic standard solution to a 1-μg/mL working solution. In five 100-mL volumetric flasks, 20 mL of a thiourea and ascorbic acid mixture and 5 mL of concentrated hydrochloric acid were added, followed by 0.1, 0.2, 0.4, 0.8, and 1 mL of the arsenic working solution, respectively. After adjusting to the final volume, the arsenic concentration was measured using a dual-channel atomic fluorescence spectrometer. For each *C. elegans* sample, arsenic content was determined by wet digestion and quantified via standard regression equations. The arsenic concentration was calculated as follows:

(1) $$\begin{array}{c} X=\frac{\left(C-C_{0}\right)\times V\times 1000}{m\times 1000\times 1000}, \end{array}$$

where *X* is the arsenic content per unit protein, mg/kg, *C* is the measured arsenic concentration in the sample solution, ng/mL, *C*_0_ is the measured arsenic concentration in the blank digestion solution, ng/mL, *V* is the total volume of the digestion solution, mL, and *m* is the total protein weight, g.

### 2.5. Fluorescence Analysis

After 24 h of exposure to sodium arsenite-OP50 mixtures, *C. elegans* were washed with M9 buffer and mounted on microscope slides. Fluorescence observations were performed using a Zeiss Axioskop 2 Plus fluorescence microscope (Carl Zeiss, Jena, Germany), and images were processed with Image J software.

### 2.6. RNA Extraction and Real-Time PCR

Total RNA was extracted from *C. elegans* using an RNA extraction kit (Vazyme, Nanjing, China). Complementary DNA (cDNA) was synthesized from RNA using the TransScript Uni All-in-One First-Strand cDNA Synthesis SuperMix for qPCR (TransGen Biotech, Beijing, China). Gene expression analysis was performed with SYBR Green qPCR Master Mix (MedChemExpress, NJ, USA) using a LightCycler 96 real-time PCR instrument (Roche Molecular Systems, Pleasanton, USA). Act-1 mRNA was used as an internal control, and the results were calculated using the 2^−∆∆Ct^ method. Primer sequences are listed in Table 1.

### 2.7. Statistical Analysis

All experiments were performed at least three times. Data were analyzed using GraphPad Prism 9 software and presented as the mean ±  standard deviation (SD). For comparisons between two groups, *t*-tests were applied, while one-way analysis of variance (ANOVA) was used for multiple group comparisons. Statistical differences in gene expression, fluorescence intensity, and avoidance behavior in *C. elegans* were assessed, with *p* < 0.05 considered statistically significant.

## 3. Results

### 3.1. Sodium Arsenite Induces Avoidance Behavior in *C. elegans*

**Figure 1:**
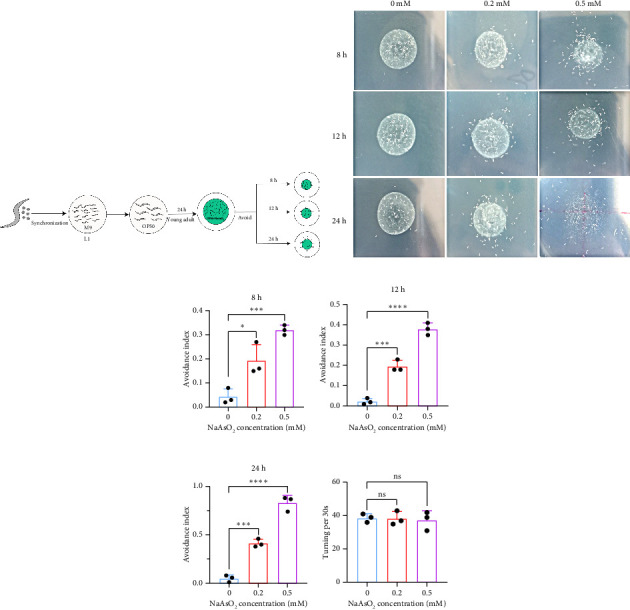
The avoidance behavior of *C. elegans* induced by sodium arsenite. (a) Schematic diagram of *C. elegans* avoidance index. (b) Schematic representation of the avoidance response of *C. elegans* exposed to 0, 0.2, and 0.5 mM sodium arsenite concentrations at 8, 12, and 24 h. (c) 8-h avoidance index. (d) 12-h avoidance index. (e) 24-h avoidance index. (f) Turning index (one-way analysis of variance was used for comparisons among multiple groups, and then the Dunnett-*t* test was used for comparison). (^∗∗∗^*p* < 0.001, ^∗∗∗∗^*p* < 0.0001, and ns *p* > 0.05).

Pathogen avoidance behavior is one of the defense strategies that protect *C. elegans* from infection and improve survival rates [15]. This phenomenon is widespread in the biological world, but does *C. elegans* exhibit similar avoidance responses to toxic compounds? In this study, we discovered for the first time that *C. elegans* displays avoidance behavior when encountering OP50 bacterial lawns containing sodium arsenite. The OP50 lawn with sodium arsenite was placed at the center of the plate. We observed that nematodes did not exhibit avoidance in an OP50-only lawn within 24 h; however, upon encountering OP50 with sodium arsenite, the avoidance index varied with different concentrations of sodium arsenite. The avoidance index increased as sodium arsenite concentration rose. The nematodes crawled away from the bacterial lawn to escape the harmful effects of sodium arsenite. We recorded the avoidance index at three time points: 8, 12, and 24 h. After 8 h of exposure, compared with the control group (0.04 ± 0.03), the avoidance index of nematodes exposed to 0.2 and 0.5 mM sodium arsenite significantly increased to 0.193 ± 0.067 (*p* < 0.05) and 0.32 ± 0.02 (*p* < 0.001), respectively. After 12 h, compared with the control group (0.02 ± 0.02), the avoidance index of nematodes exposed to 0.2 and 0.5 mM sodium arsenite also significantly increased to 0.197 ± 0.029 (*p* < 0.001) and 0.38 ± 0.03 (*p* < 0.0001), respectively. After 24 h, compared with the control group (0.05 ± 0.03), the avoidance index of nematodes exposed to 0.2 and 0.5 mM sodium arsenite significantly rose to 0.413 ± 0.042 (*p* < 0.001) and 0.83 ± 0.078 (*p* < 0.0001), respectively. To determine whether the nematode's movement influenced the avoidance index, we measured the body bend frequency over 30 s. Compared with the control group (38.67 ± 2.52), the body bend frequency in nematodes exposed to 0.2 and 0.5 mM sodium arsenite was 38.33 ± 4.16 and 37.33 ± 5.69, respectively, showing no significant difference (Figure 1). This indicates that sodium arsenite exposure does not affect nematode locomotion. In summary, when exposed to sodium arsenite, *C. elegans* perceives the harm and increases avoidance behavior as an adaptive response to the environment.

### 3.2. Arsenic Content Increases With Sodium Arsenite Treatment Concentration in *C. elegans*

**Figure 2:**
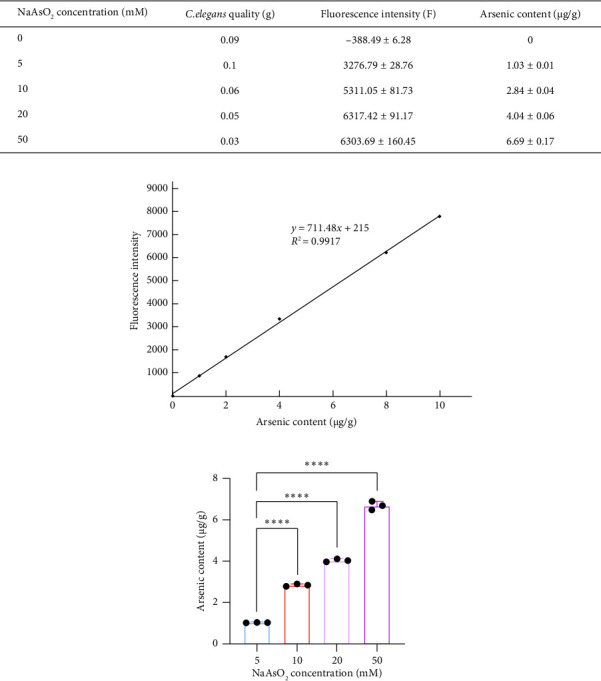
The concentration of sodium arsenite in *C. elegans* increases with higher exposure concentrations. (a) Arsenic content in *C. elegans* after treatment with different concentrations of sodium arsenite. (b) Fluorescence intensity versus arsenic content standard curve. (c) Analysis of the difference in arsenic content in *C. elegans* after treatment with different concentrations of sodium arsenite (one-way analysis of variance was used for comparisons among multiple groups, and then the Dunnett-*t* test was used for comparison). (^∗∗∗∗^*p* < 0.0001).

How does the arsenic content in *C. elegans* change during sodium arsenite exposure? We added sodium arsenite at concentrations of 0, 5, 10, 15, and 20 mM to the nematodes' food source (OP50) and measured the arsenic content in *C. elegans* after 24 h. First, the atomic fluorescence spectrometer was calibrated to optimal conditions, and fluorescence intensities of the standard series were measured. Using arsenic concentration as the *x*-axis and fluorescence intensity (with the baseline fluorescence subtracted) as the *y*-axis, we plotted a standard curve. Under the same conditions, we measured the fluorescence intensity of the samples and sample blanks. Sample fluorescence intensity minus blank fluorescence intensity was used to calculate arsenic content through the standard regression equation. The mass of nematodes was accurately determined after removing moisture. The fluorescence intensities for *C. elegans* exposed to 0, 5, 10, 20, and 50 mM sodium arsenite were −388.49 ± 6.28, 3276.79 ± 28.76, 5311.05 ± 81.73, 6317.42 ± 91.17, and 6303.69 ± 160.45, respectively. By applying these values to the linear regression equation and calculating according to the formula, the arsenic content in nematodes was found to be 0 μg/g, 1.03 ± 0.01 μg/g, 2.84 ± 0.04 μg/g, 4.04 ± 0.06 μg/g, and 6.69 ± 0.17 μg/g, respectively. Compared with the 5-mM group, the differences in the arsenic content in the 10, 20, and 50-mM groups were statistically significant (*p* < 0.0001) (Figure 2). This indicates that the arsenic content in *C. elegans* increases with the concentration of sodium arsenite.

### 3.3. Sodium Arsenite Promotes the Avoidance Behavior of *C. elegans* Through TPH-1

**Figure 3:**
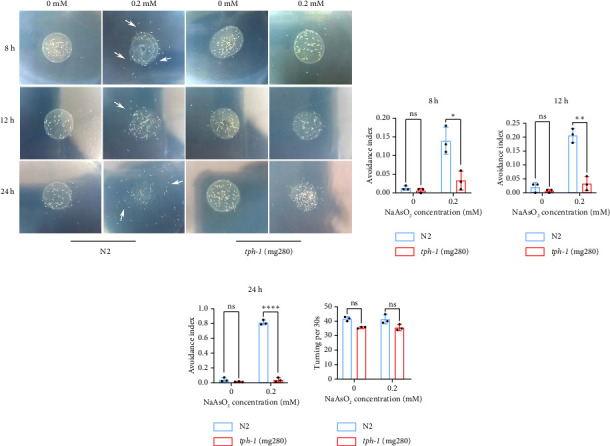
Sodium arsenite promotes escape behavior in *C. elegans* via TPH-1. (a) Escape behavior of N2 and *tph-1*(mg280) *C. elegans* exposed to 0 and 0.2-mM sodium arsenite for 8, 12, and 24 h. (b) 8-h avoidance index. (c) 12-h avoidance index. (d) 24-h avoidance index. (e) Turning index (the comparison between the two groups was conducted using Student's *t*-test). (^∗^*p* < 0.05, ^∗∗^*p* < 0.01, ^∗∗∗∗^*p* < 0.0001, and ns *p* < 0.05).

When *C. elegans* is exposed to the pathogen *P. aeruginosa PA14*, the expression of TPH-1 in ADF neurons increases, synthesizing serotonin, which enhances the nematode's learning ability and reverses its preference behavior toward PA14 [14]. Further studies have shown that TPH-1 synthesized in ADF neurons binds to MOD-1 receptors in the interneurons AIZ and AIY, activating downstream signaling pathways that regulate olfactory learning behavior toward PA14 odors [16]. TPH-1 plays a critical role in modulating learned avoidance. We aimed to determine whether TPH-1 is involved in regulating arsenite-induced avoidance behavior by knocking out *tph-1* in *C. elegans* and examining changes in the avoidance index. After exposing *tph-1* knockout mutants to 0-mM sodium arsenite for 8, 12, and 24 h, no significant difference in the avoidance index was observed compared with N2 wild-type worms. However, after 8 h of exposure to 0.2-mM sodium arsenite, the avoidance index of *tph-1* knockout mutants (0.03 ± 0.03) was significantly lower than that of N2 worms (0.11 ± 0.03) (*p* < 0.05). After 12 h, the avoidance index of *tph-1* mutants (0.03 ± 0.01) was significantly reduced compared with N2 worms (0.21 ± 0.02) (*p* < 0.01). After 24 h, the avoidance index of *tph-1* mutants (0.03 ± 0.03) was markedly lower than that of N2 worms (0.81 ± 0.03) (*p* < 0.001) (Figure 3). These results indicate that knocking out TPH-1 significantly reduces the avoidance response, suggesting that TPH-1 plays a central role in the regulation of avoidance behavior.

### 3.4. The Expression of TPH-1 in ADF Neurons Is Upregulated Upon Exposure to Sodium Arsenite

**Figure 4:**
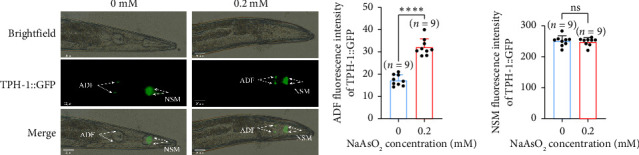
The effect of sodium arsenite on the expression of TPH-1 in ADF and NSM neurons of *C. elegans*. (a) Expression of TPH-1 in ADF neurons of *C. elegans* after exposure to sodium arsenite. (b) Quantitative analysis of ADF fluorescence intensity. (c) Quantitative analysis of NSM fluorescence intensity (the comparison between the two groups was conducted using Student's *t*-test). (^∗∗∗∗^*p* < 0.0001 and ^ns^*p* > 0.05).

In *C. elegans*, key genes involved in serotonin synthesis include *tph-1*, *cat-1*, and *cat-2*, with *tph-1* being essential for serotonin synthesis [17]. Studies have shown that *tph-1* is primarily expressed in ADF and NSM neurons. By observing *tph-1*::GFP expression, we found that in *C. elegans* exposed to 0.2 mM sodium arsenite, the fluorescence intensity of *tph-1*::GFP in ADF neurons was significantly increased compared to the control, whereas the fluorescence in NSM neurons showed no significant change (Figure 4). These results indicate that sodium arsenite exposure increases *tph-1* expression in ADF neurons, leading to enhanced serotonin synthesis.

### 3.5. Immune Pathways Are Required for Response to Sodium Arsenite Exposure

**Figure 5:**
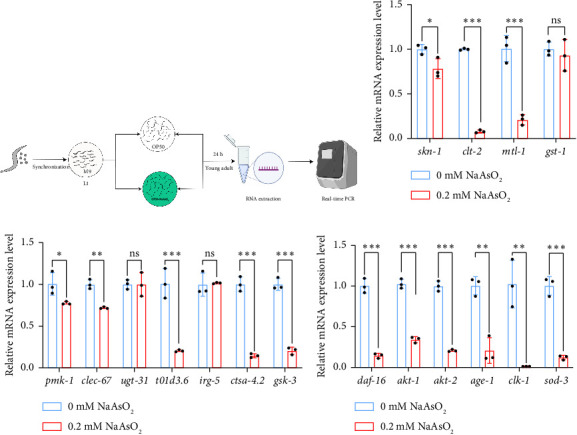
The effect of sodium arsenite exposure on the expression of immune genes in *C. elegans*. (a) Schematic diagram of *C. elegans* infection and Q-PCR. (b) Effect of sodium arsenite on the SKN-1 pathway in *C. elegans*. (c) Effect of sodium arsenite on the PMK-1 pathway in *C. elegans*. (d) Effect of sodium arsenite on the DAF-16 pathway in *C. elegans*. (the comparison between the two groups was conducted using Student's *t*-test). (^∗^*p* < 0.05, ^∗∗^*p* < 0.01, ^∗∗∗^*p* < 0.001, ^∗∗∗∗^*p* < 0.0001, and ^ns^*p* > 0.05).

**Figure 6:**
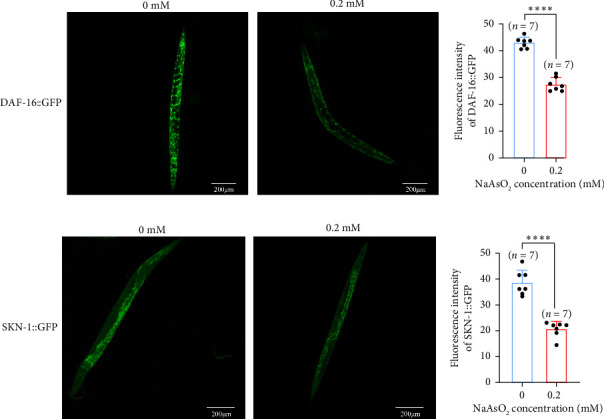
Reduction in DAF-16 and SKN-1 fluorescence in *C. elegans* after exposure to sodium arsenite. (a) Effect of sodium arsenite exposure on DAF-16 expression in *C. elegan*s. (b) Quantitative analysis of DAF-16 fluorescence intensity. (c) Effect of sodium arsenite exposure on SKN-1 expression in *C. elegans*. (d) Quantitative analysis of SKN-1 fluorescence intensity (the comparison between the two groups was conducted using Student's *t*-test). (^∗∗∗∗^*p* < 0.0001).

We next examined the expression of immune-related genes in *C. elegans* following exposure to sodium arsenite. After exposure to 0.2-mM sodium arsenite, we observed a significant downregulation of the following genes in the DAF-16 pathway: *daf-16, akt-1, akt-2, age-1, clk-1, and sod-3*. In the SKN-1 pathway, *skn-1, ctl-2*, and *mtl-1* were significantly downregulated, while the expression of *gst-1* showed no significant change. In the PMK-1 pathway, *pmk-1, clce-67, t01d3.6, ctsa-4.2*, and *gsk-3* were significantly downregulated, whereas the expression of *ugt-31* and *irg-5* remained unchanged (Figure 5). Furthermore, we observed a significant decrease in the fluorescence intensity of DAF-16 and SKN-1 following exposure to 0.2-mM sodium arsenite (Figure 6). These findings collectively suggest that exposure to sodium arsenite suppresses the immune system of *C. elegans.*

## 4. Discussion

This study is the first to systematically reveal the avoidance behavior and potential molecular mechanisms of *C. elegans* exposed to sodium arsenite. This behavior appears to be an ecological adaptation strategy aimed at protecting nematodes from the toxic effects of arsenic contamination in the environment. Our results show that exposure to sodium arsenite induces significant avoidance behavior through upregulation of the serotonin synthesis gene *tph-1*. Elevated *tph-1* expression enables *C. elegans* to produce more serotonin, activating neural pathways associated with avoidance behavior to cope with adverse environmental conditions. Using *C. elegans* as a model, this study not only uncovers the direct toxic effects of arsenic pollution on soil organisms but also explores the behavioral adaptations of nematodes in response to arsenic exposure.

### 4.1. Ecological Adaptation Significance of Avoidance Behavior

*C. elegans* is a free-living nematode widely distributed in soil, frequently exposed to complex microenvironments that contain a variety of nutrients, environmental toxins, and pathogenic microorganisms [18]. Avoidance behavior is an important adaptive response in such dynamic environments [19]. Our study demonstrates that *C. elegans* shows a concentration-dependent avoidance of arsenic-contaminated areas when exposed to sodium arsenite, with the avoidance index increasing as sodium arsenite concentration rises. This behavior can be viewed as an active defense strategy that helps reduce arsenic exposure risks, enhancing survival. In nature, soil arsenic contamination primarily results from geological processes, industrial emissions, and agricultural activities [20]. By avoiding toxic areas, *C. elegans* may also indirectly influence the distribution of other soil organisms and regulate microbial community dynamics. This ecological adaptation may be evolutionarily significant, allowing nematodes to maintain an ecological advantage in fluctuating, complex, and potentially toxic environments [14].

### 4.2. Serotonin-Regulated Behavioral Mechanism

This study further elucidates the critical role of serotonin in arsenic-induced avoidance behavior. Deletion of the serotonin synthesis gene *tph-1* led to a significant reduction in avoidance response, indicating the central role of *tph-1* in regulating this behavior. According to previous reports, the upregulation of *tph-1* expression in ADF neurons leads to serotonin secretion, which is then received by MOD-1 receptors in AIY and AIZ neurons, transmitting the signal to regulate avoidance behavior [21]. This finding suggests that serotonin plays a crucial role in the neuroregulation of *C. elegans*'s behavioral adaptation to environmental toxins [22]. Whether *C. elegans* utilizes these interneurons to modulate avoidance in response to sodium arsenite remains to be further investigated, this serotonin-mediated avoidance response can be considered an adaptive neurobehavioral response in a diverse environment. Serotonin not only regulates avoidance behavior in nematodes but also likely interacts with other neurotransmitters to modulate sensory perception and decision-making, enabling *C. elegans* to rapidly respond upon detecting toxic chemicals [23]. This rapid sensory response allows nematodes to optimize survival strategies in highly dynamic microhabitats, conferring an evolutionary advantage. The NSM neurons, located in the pharyngeal region of *C. elegans*, play a key role in sensing food and regulating feeding behavior, primarily through the release of serotonin (5-HT) to promote pharyngeal pumping [24]. While NSM is essential for feeding, it is not a major driver of avoidance behavior. Previous studies have indicated that serotonin released from NSM has a minimal impact on behavior, likely due to its low levels being insufficient to activate MC motor neurons in the pharynx, which are required for initiating feeding in response to familiar food. In contrast, serotonergic signaling from the ADF neurons may serve as a more potent endocrine cue, systemically regulating feeding activation and orchestrating a range of behavioral and physiological adaptations [25, 26].

### 4.3. Neuro-Immune Interaction Mechanism

In addition to neural regulation, the immune system plays an important role in arsenic exposure [27]. This study also found that the innate immune system of *C. elegans* was compromised under sodium arsenite exposure. Specifically, signaling pathways such as PMK-1, SKN-1, and DAF-16 were downregulated after arsenic exposure. This indicates a neuro-immune interaction mechanism by which nematodes can manage toxic environments, prioritizing avoidance behavior as an adaptive strategy. This neuro-immune interaction mechanism holds a significant scientific value for understanding the response of organisms to environmental toxic stress. Future studies could further investigate the synergistic roles of the nervous and immune systems under various environmental stressors.

### 4.4. Broad Ecological Impact of Arsenic Pollution

Arsenic pollution is widespread globally, especially in soils and groundwater, posing severe threats to ecosystems and human health. As a key species within the ecosystem, *C. elegans* provides insights into the multidimensional biological effects of arsenic pollution through its behavioral responses and physiological adaptations [28]. The nematode's neuro-immune responses to arsenic toxicity not only offer a new perspective on the ecological toxicity of arsenic but also provide a scientific basis for ecological risk assessments of environmental pollution. The ecological risks of arsenic pollution primarily involve potential disruptions to ecosystem functions and biodiversity [29]. The sensitivity of *C. elegans* to arsenic pollution could serve as a biological marker for environmental monitoring, with its avoidance behavior reflecting changes in environmental arsenic concentrations, thus providing a reliable reference for pollution monitoring and risk assessment. Future research could utilize *C. elegans* as a bioindicator, combining its avoidance behavior and immune responses to gain a better understanding of the ecological impacts of arsenic pollution.

## 5. Conclusions and Future Directions

Although this study reveals the ecological adaptation mechanisms of *C. elegans* under arsenic exposure, there are limitations. First, this study primarily focuses on the effects of sodium arsenite on avoidance behavior, without considering the potential differences in behavior and physiology induced by other arsenic compounds (such as arsenate). Additionally, different concentrations and exposure methods in varying environments may influence the adaptation mechanisms of *C. elegans*, warranting further exploration of nematode strategies in different arsenic-contaminated environments. Future research could extend to other model organisms or wild populations to further examine the ecological effects of arsenic pollution. For example, comparative studies of avoidance behavior in *C. elegans* and other soil organisms (such as earthworms and insects) could reveal the broader impacts of arsenic within ecosystems. Moreover, by analyzing the physiological and genetic effects of arsenic pollution on the offspring of *C. elegans*, we could gain a deeper understanding of the intergenerational effects of environmental toxins and their long-term impacts on ecosystems.

## Figures and Tables

**Table 1:** Q-PCR primer sequences.

Primer	Forward	Reverse
pmk-1	TCATCCGACTCCACGAGAAG	CATTCAGCAGCACAAACAGTTC
clec-67	CCCTTCCTATTTGGCTGTT	ATCCGTTATCCCAGGTCA
irg-5	CGCTGTCAGAAAGACGCTT	TGAAGAATGTTCCAGTTGCC
f01d5.5	CCTTCTGCCCAGTAACCTGT	TTTCACCCAGTTGACGCA
ugt-31	TAATGCTCCGATGTTGGC	GCAGATTTGCGAGGGTTT
skn-1	TCATCACCAACATCCATACG	CAACTGCTTGAAGACTGTCG
t01d3.6	TCAATCTGTGTGGCTTGG	AGTAGTAAGGGATGGGCGT
ctsa-4.2	GGGCAAGTCACAGAATACG	CTGGAACATTTGGAAGGTCT
daf-16	CGGGAGAGAGGGACACGCTTC	ACGGAATTGCTCAGCCACCATG
akt-2	CACCACAATACGCCCCACACTC	AGGACCTCGCGCCACTATAGC
age-1	CGCCACGGCAACATCCTCAG	GGCTGCTCAATCGCCAACTCC
sod-3	GGCTAAGGATGGTGGAGAAC	ACAGGTGGCGATCTTCAAG
clk-1	GCACATACTGCTGCTTCTCG	TCATTCCATCGTGTTCTACTCC
ctl-2	ACACGGACACGCATTACCA	TTCCTCCAAACAGCCACC
mtl-1	CGGAGACAAATGTGAATGC	AGTTCCCTGGTGTTGATGG
gst-1	AACCTATGAACAATGGGCTG	TAAGACGAGCGAGATGACG
akt-1	GGGATGGCTTCACAAGAAAG	ATGGCTCAGGAAACGGTT
gsk-3	GCAGTTACTATCGTGCTCGCT	GCAAGGAATACCACTCCGA
act-1	TCGGTATGGGACAGAAGGAC	CATCCCAGTTGGTGACGATA

## Data Availability

The data that support the findings of this study are available from the corresponding authors upon reasonable request.
